# A T-cell-related signature for prognostic stratification and immunotherapy response in hepatocellular carcinoma based on transcriptomics and single-cell sequencing

**DOI:** 10.1186/s12859-023-05344-7

**Published:** 2023-05-25

**Authors:** Xu Chen, Chuang Peng, Yu Chen, Bai Ding, Sulai Liu, Yinghui Song, Yuhang Li, Bo Sun, Ranzhiqiang Yang

**Affiliations:** 1 Department of Hepatobiliary Surgery, Hunan Provincial People’s Hospital, The First Affiliated Hospital of Hunan Normal University, Changsha, 410005 Hunan China; 2 Central Laboratory of Hunan Provincial People’s Hospital, The First Affiliated Hospital of Hunan Normal University, Changsha, 410005 Hunan China

**Keywords:** T-cell marker genes, Single-cell RNA sequencing, Immunotherapy, Hepatocellular carcinoma, Prognostic signature

## Abstract

**Background:**

Hepatocellular carcinoma (HCC) is the fifth most frequently diagnosed malignancy and the third leading cause of cancer death globally. T cells are significantly correlated with the progression, therapy and prognosis of cancer. Limited systematic studies regarding the role of T-cell-related markers in HCC have been performed.

**Methods:**

T-cell markers were identified with single-cell RNA sequencing (scRNA-seq) data from the GEO database. A prognostic signature was developed with the LASSO algorithm in the TCGA cohort and verified in the GSE14520 cohort. Another three eligible immunotherapy datasets, GSE91061, PRJEB25780 and IMigor210, were used to verify the role of the risk score in the immunotherapy response.

**Results:**

With 181 T-cell markers identified by scRNA-seq analysis, a 13 T-cell-related gene-based prognostic signature (TRPS) was developed for prognostic prediction, which divided HCC patients into high-risk and low-risk groups according to overall survival, with AUCs of 1 year, 3 years, and 5 years of 0.807, 0.752, and 0.708, respectively. TRPS had the highest C-index compared with the other 10 established prognostic signatures, suggesting a better performance of TRPS in predicting the prognosis of HCC. More importantly, the TRPS risk score was closely correlated with the TIDE score and immunophenoscore. The high-risk score patients had a higher percentage of SD/PD, and CR/PR occurred more frequently in patients with low TRPS-related risk scores in the IMigor210, PRJEB25780 and GSE91061 cohorts. We also constructed a nomogram based on the TRPS, which had high potential for clinical application.

**Conclusion:**

Our study proposed a novel TRPS for HCC patients, and the TRPS could effectively indicate the prognosis of HCC. It also served as a predictor for immunotherapy.

**Supplementary Information:**

The online version contains supplementary material available at 10.1186/s12859-023-05344-7.

## Introduction

Hepatocellular carcinoma (HCC) is the fifth most frequently diagnosed malignancy and the third leading cause of cancer death globally [1]. A total of 905,677 million cases were estimated to be first diagnosed with HCC, and this disease caused 830,180 deaths in 2020 globally, ranking for approximately 4.7% of newly diagnosed cancer cases and 8.3% of cancer-related deaths [2]. Characterized by a high rate of invasiveness, recurrence and metastasis, the prognosis of HCC is poor, and the 5-year overall survival (OS) is only approximately 30% [3]. Although some risk factors have been identified for HCC, including HBV infection and alcohol consumption, the molecular mechanism of HCC is far from being elucidated [4]. Although chemotherapy, targeted therapy and immunotherapy have been used for the treatment of HCC, limited biomarkers could be used for the response to these therapies and prognosis. Increasing evidence has emerged for novel biomarkers in the prognosis and therapy response of HCC.

The tumor microenvironment (TME) denotes the presence of noncancerous cells and tumor-related components, including molecules produced and released by them [5]. The crosstalk between the infiltrated immune cells in the tumor microenvironment and tumor plays a vital role in the multistep progression of cancer [6]. As a vital element of the tumor microenvironment, T cells are significantly correlated with the progression, therapy and prognosis of cancer [7, 8]. T cells are key mediators of tumor destruction, and their specificity for tumor-expressed antigens is of paramount importance [9]. The absence of T cells could lead to tumor immune escape and treatment failure [10]. Moreover, T-cell-related markers could serve as prognostic biomarkers for various types of cancer, including lung squamous cell carcinoma [10], renal cell carcinoma [11], and uveal melanoma [12]. Thus, it is necessary to explore the prognostic value of T cells and their association with therapy response in HCC.

Single-cell RNA sequencing (scRNA-seq) has provided a good approach for understanding the TME and immunotherapy [13]. Integrated analysis of single-cell and bulk RNA sequencing was also provided as a new way to identify prognostic biomarkers and therapeutic targets for cancer [14–18]. Herein, an integrative analysis of scRNA-seq and bulk RNA-seq of HCC was performed to identify T-cell marker genes and develop a prognostic signature, which could be used for prognostic stratification and immunotherapy response in HCC. Our results may provide more evidence for prognostic markers and therapeutic targets for HCC.

## Materials and methods

### scRNA-seq data and transcriptome data acquisition

All the datasets used in this study are public/open access datasets. The scRNA-seq data of HCC tumor samples were downloaded from the GSE162616 dataset via the GEO database (https://www.ncbi.nlm.nih.gov/geo/). The details of scRNA-seq data of HCC tumor samples are provided in Additional file 1: Table S1. The bulk transcriptome RNA-seq data and corresponding clinical data of HCC (n = 371) were obtained from The Cancer Genome Atlas (TCGA, https://portal.gdc.cancer.gov/). We chose the GSE14520 (n = 221) dataset as the test cohort for the validation of the subtype and prognostic signature. The clinical information of TCGA and GSE14520 were shown in Additional file 2: Table S2. Those cases histologically diagnosed with HCC with valid information about age, gender and overall survival were included in our study while those cases that were metastatic HCC were excluded from our study. Another three eligible immunotherapy datasets, GSE91061 (n = 39, anti-CTLA4 and anti-PD1 therapy), PRJEB25780 (n = 78, anti-PD1 therapy) and IMigor210 (n = 298, anti-PD1 therapy), were used to verify the role of the risk score in immunotherapy response.

### Single-cell RNA-seq analysis

The scRNA-seq data were processed with the Seurat R package (version 4.0), an R toolkit for single-cell genomics [19]. Genes detected in fewer than 3 cells and cells with fewer than 50 detected genes were excluded, and the mitochondrial proportion was limited to less than 5%. After data normalization with the LogNormalize method, we performed principal component analysis (PCA) and UMAP analysis for unsupervised clustering, with which we could visualize cell populations on a two-dimensional map [20]. Cell annotation was performed using the SingleR package with reference data from the Human Primary Cell Atlas [21]. To identify marker genes of each cluster, we selected the “FindAllMarkers” function and fold change (FC) ≥ 1 and the minimum cell population fraction in either of the two populations of 0.4 as threshold values. T-cell-related markers were defined as the markers of corresponding T-cell clusters.

### Genetic mutation and prognostic value analysis

The single nucleotide variation (SNV) and copy number variation (CNV) atlas of T-cell-related markers was generated with GSCALite, a web tool for gene set cancer analysis based on the TCGA dataset [22]. Seven types of mutation were included in this analysis: Missense_Mutation, Nonsense_Mutation, Frame_Shift_Ins, Splice_Site, Frame_Shift_Del, In_Frame_Del, In_Frame_Ins. CNV nanlysis was processed through GISTIC2.0 [23]. To identify T-cell-related markers with prognostic significance in HCC, univariate Cox regression analysis was performed.

### Nonnegative matrix factorization (NMF) clustering

To explore whether T-cell-related markers with prognostic significance could distinguish different types of HCC, we performed NMF clustering with the nmf R package. Genes with a median absolute deviation (MAD) value > 0.5 were chosen for sample clustering. A cluster heatmap was generated with the “pheatmap” package. The survival curve of different clusters was drawn with the Kaplan‒Meier method.

### Development and validation of the prognostic signature and predictive nomogram

Based on T-cell-related markers with prognostic significance in HCC, we conducted least absolute shrinkage and selection operator (LASSO) analysis via the glmnet R package to identify candidates for the prognostic signature. Based on the coefficient value of each candidate, we calculated the risk score of each HCC sample. After that, we could distinguish HCC samples into high- and low-risk groups with the medium value as the cutoff. The clinical outcome of different HCC groups was analyzed with the log-rank test. The survivalROC and rms R packages were applied to construct a time‐dependent ROC curve and C-index to evaluate the predictive power of the prognostic signature. Moreover, all independent prognostic risk factors were identified with univariate and multivariate Cox analyses. We then compared our signature with 10 other prognostic signatures that have been developed for HCC. More specifically, a total of 468 items about prognostic signature for HCC were identified by searching for “TCGA” and “prognostic signature” AND “HCC” in Pubmed (https://pubmed.ncbi.nlm.nih.gov/) on December 25, 2022. We used Excel to generate 10 random numbers from 1 to 468, and these 10 random numbers corresponding to the items were selected for further comparison with our prognostic signature. This was followed by the construction of a predictive nomogram based on all independent prognostic risk factors to predict the 1-, 3-, and 5-year overall survival of HCC patients. Decision curve analysis (DCA) was performed using the ggDCA R package to evaluate the potential of the predictive nomogram for clinical application.

### Immune landscape, gene set enrichment and therapeutic response analysis

The ESTIMATE method was used to explore the TME score (immunoscore, stromascore and ESTIMATEScore) of HCC [24]. The abundance of immune cells in HCC was estimated with the CIBERSORT method [25]. The “ggpubr” or “vioplot” R package was used to compare the expression of human leukocyte antigen (HLA)-related genes and immune checkpoints in different groups. To clarify the potential mechanisms of the high- and low-risk groups, we conducted GSEA using gene sets of c2kegg, and the threshold value was normalized enrichment score (NES)|> 1 and nominal (NOM) p value < 0.05. The tumor immune dysfunction and exclusion (TIDE) score and immunophenoscore (IPS) were used to evaluate the performance of the prognostic signature in the immune response [26]. These two scores could guide doctors in selecting patients who are more suitable for immune checkpoints. Moreover, the oncoPredict R package was used to predict drug sensitivity to common chemotherapy and targeted therapy drugs. Drug sensitivity was obtained from Genomics of Drug Sensitivity in Cancer (https://www.cancerrxgene.org/). Drug sensitivity was detected by measuring the area under the concentration‒response curve value, and a high AUC indicated low sensitivity.

## Results

### Single-cell analysis reveals cell subtypes and T-cell-related markers

**Fig. 1:**
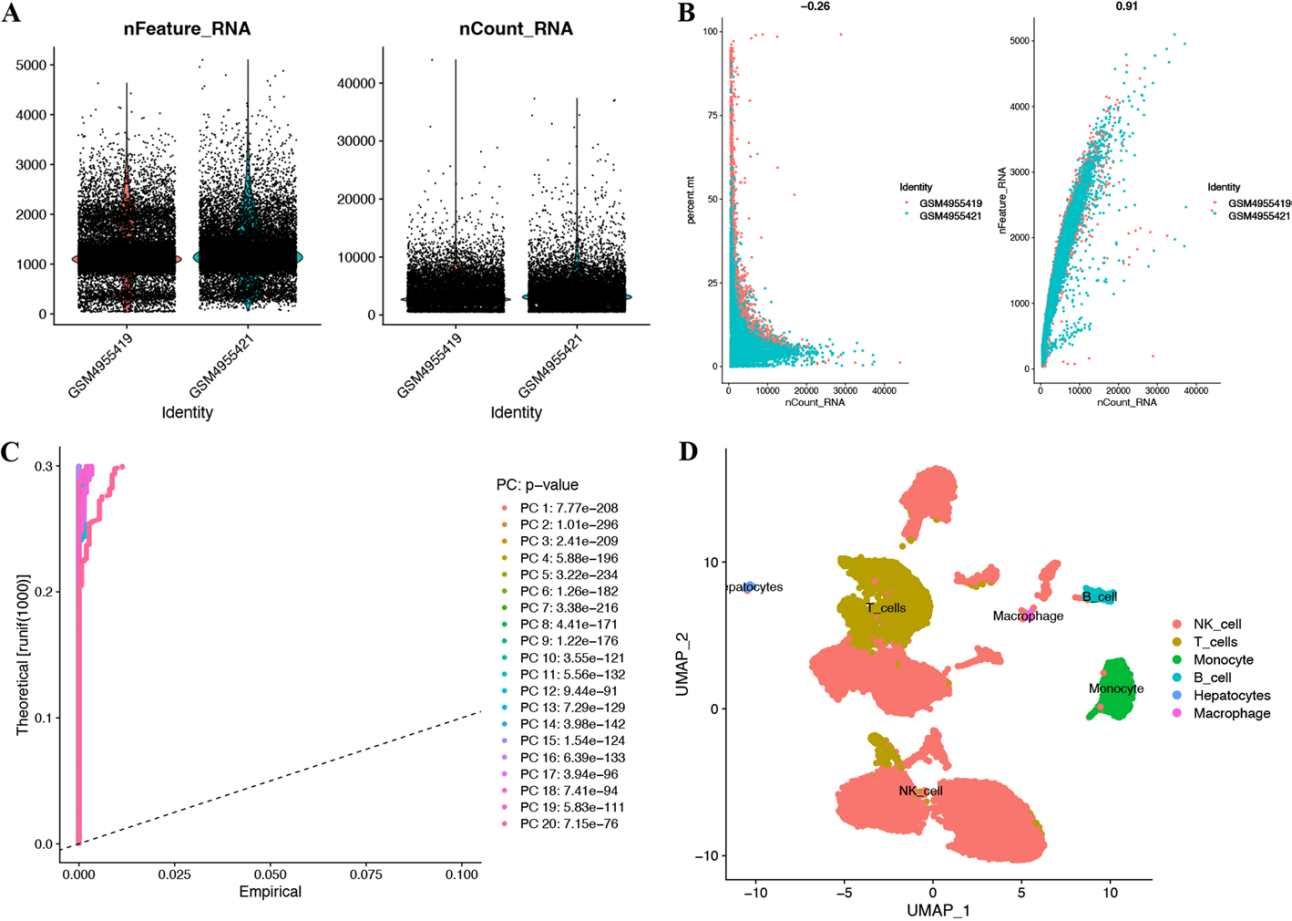
Analysis of single-cell RNA sequencing from two HCC samples. **A** Post quality control filtering of each sequenced cell. **B** Correlation analysis between nFeature and nCount. **C** A total of 20 clusters of all cells were identified. **D** The UMAP dimensionality reduction algorithm identified 6 subtypes of cells

HCC scRNA-seq data were preprocessed with stringent quality control metrics, and we obtained 34,170 high-quality cell samples from two HCC tissues (Fig. 1A). There was a strong positive correlation between the number of genes detected and the sequencing depth, with a Pearson correlation coefficient of 0.91 (Fig. 1B). These samples could be divided into 17 clusters (Fig. 1C). Using the UMAP technique to annotate acknowledged cell types, we obtained a total of 6 types of cells, including NK cells, T cells, monocytes, B cells, hepatocytes, and macrophages (Fig. 1D). Moreover, we also obtained 181 T-cell-related genes (Fig. 1D, Additional file 3: Table S3).

### The genetic mutation atlas of T-cell-related genes in HCC.

The CNV landscape of T-cell-related genes in HCC is presented in Additional file 4: Figure S1, revealing that more than half of T-cell-related genes had widespread CNV amplification, while DOK2, DUSP4, SARAF, LEPROTL1 and NSD3 had a significant homozygous deletion. SNV analysis suggested that ANKRD12 ranked the highest frequency of SNVs, and missense mutations were the most common variant classification (Additional file 5: Figure S2A-2B).

### NMF identifies two subtypes in HCC

**Fig. 2:**
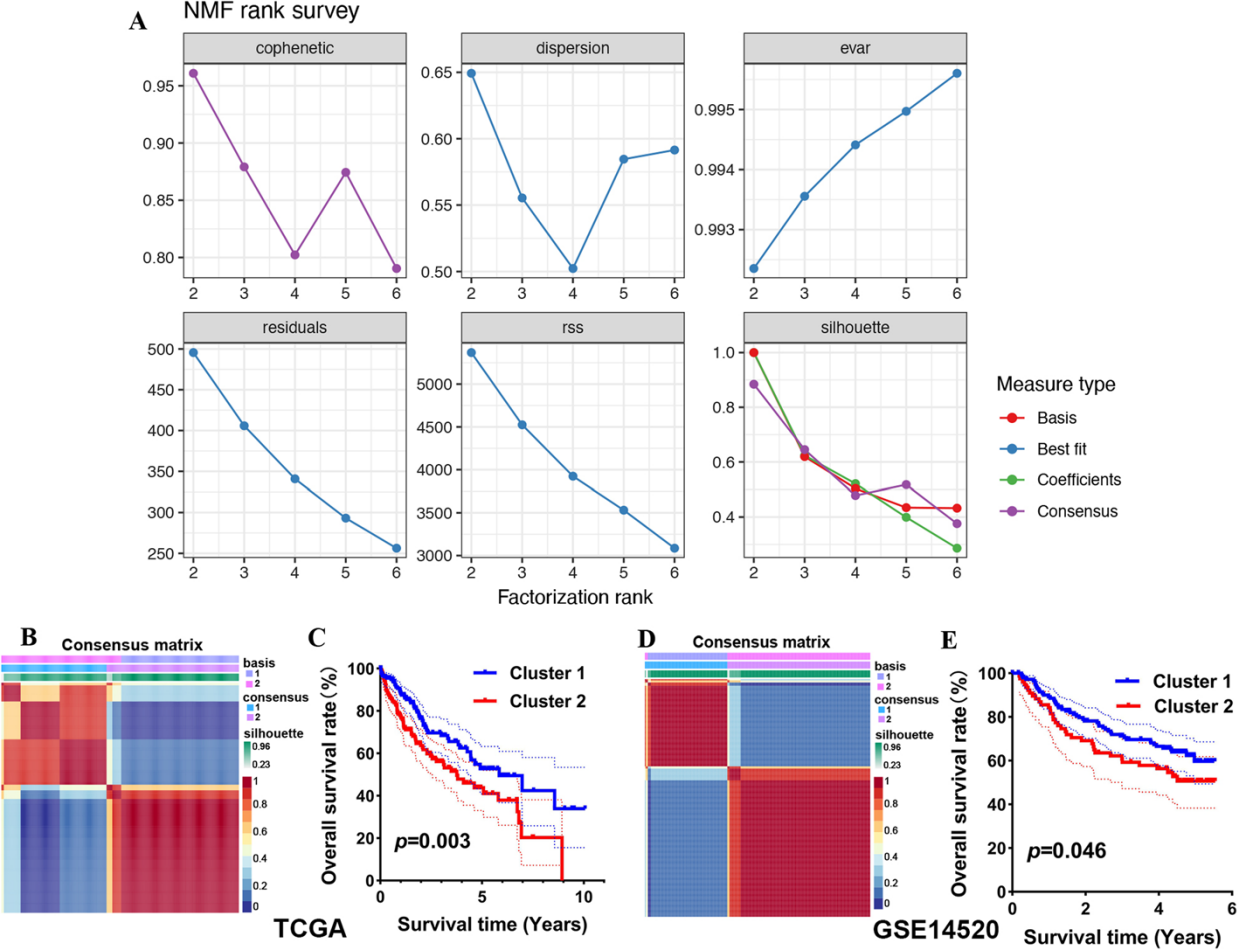
Nonnegative matrix factorization (NMF) identifies two subtypes in HCC. **A** Cophenetic correlation from NMF analysis of HCC tumors. **B** Heatmap displaying consensus clustering with robust classification in the TCGA cohort (k = 2). **C** Overall survival curve of the two clusters in the TCGA cohort. **D–E** Heatmap and overall survival curve of the two clusters in the GSE14520 cohort

A total of 55 T-cell-related genes with significant prognostic value were screened out using Cox univariate analysis (Additional file 5: Figure S2C). To clarify whether these 55 T-cell-related genes could cluster HCC into subtypes, we performed NMF analysis (Fig. 2A). As a result, HCC samples were divided into two distinct modification pattern clusters, including 183 cases in cluster C1 and 117 cases in cluster C2 (Fig. 2B). Further results showed that HCC patient cluster 2 was correlated with poor OS compared with HCC patients in cluster 1 (Fig. 2C, p = 0.003). We also verified our result using the GSE14520 cohort, and similar results were obtained (Fig. 2D–E).

### Construction and validation of the T-cell-related prognostic signature (TRPS) for HCC

**Fig. 3:**
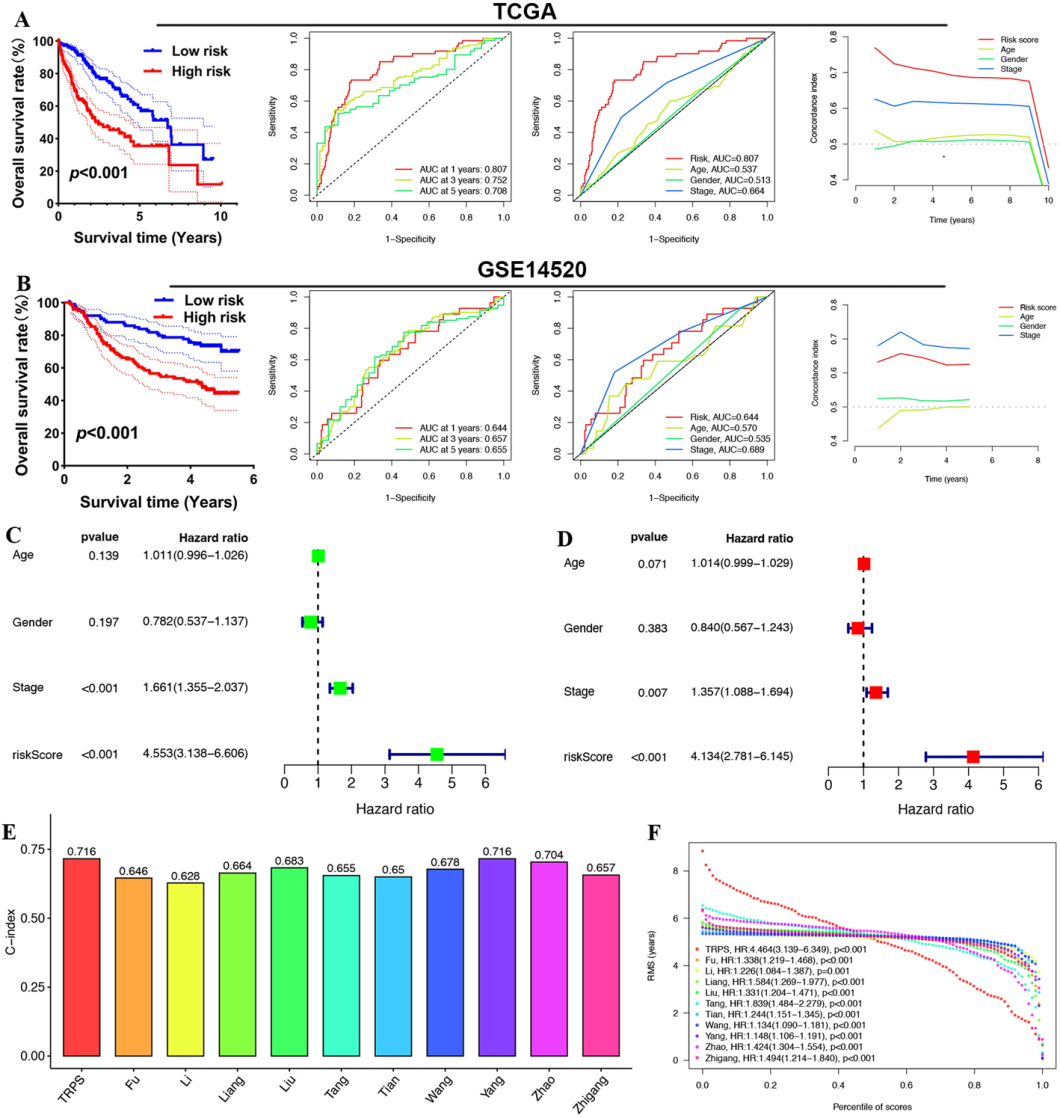
Construction and validation of a T-cell-related prognostic signature (TRPS) for HCC. **A** Survival curve, ROC curve, and C-index of TRPS in the TCGA cohort. **B** Survival curve, ROC curve, and C-index of TRPS in the GSE14520 cohort. **C–D** Univariate and multivariate Cox regression considering the risk score and clinical characteristics in the TCGA cohort. The C-index (**E**) and RMS curve (**F**) of TRPS and the other eight developed risk models

As 55 T-cell-related genes could cluster HCC into two subtypes with distinct prognoses, we then constructed a prognostic signature based on these genes. After performing LASSO regression analysis, a total of 13 T-cell-related genes were selected for the prognostic signature, and the coefficient of the candidate gene is shown in Additional file 6: Figure S3A-3B. The risk score of each HCC sample was calculated with the following formula: Risk score = ( − 0.197070704 * expression of IL7R) + (0.032065529 * expression of BATF) + (0.06105135 * expression of PRDX1) + (0.02338793 * expression of HSPA8) + (0.114580331 * expression of AHSA1) + (0.062159747 * expression of RGS2) + (0.037404701 * expression of DYNLL1) + (0.282509962 * expression of CHORDC1) + (0.109884375 * expression of NUDC) + (0.054986533* expression of OAZ1) + ( − 0.073910694 * expression of PER1) + (0.211991659 * expression of ZC3HAV1) + (0.058564557 * expression of CDV3). HCC cases could be divided into low- and high-risk groups in light of their median risk score in the training (TCGA) cohort and test (GSE14520) cohort. The number of deaths in the high-risk group was much greater than that in the low-risk group in the training cohort and test cohort (Additional file 6: Figure S3C-3D). Kaplan‒Meier curves suggested that the high-risk group was correlated with unfavorable survival outcomes versus the low-risk group in the TCGA cohort (Fig. 3A, p < 0.001). Further ROC analysis suggested that the AUCs at 1 year, 3 years, and 5 years were 0.807, 0.752, and 0.708, respectively, demonstrating the good performance of this prognostic in risk evaluation in HCC (Fig. 3A). We also compared the predictive value of this TRPS with other clinical parameters. Interestingly, the ROC curve and C-index suggested that TRPS had the best performance in risk evaluation in HCC compared with age, sex and clinical stage (Fig. 3A). To verify our results, we also verified these results using the GSE14520 dataset. HCC patients with a high risk score had a poor OS rate, with AUCs at 1 year, 3 years, and 5 years of 0.644, 0.657, and 0.655, respectively (Fig. 3B). However, the performance of TRPS in risk evaluation in HCC was better than that of age and sex but not clinical stage (Fig. 3B), which was different from the results of the TCGA dataset. Thus, it would be better to verify these results using more datasets. Moreover, univariate and multivariate Cox analyses suggested TRPS and clinical stage as independent risk factors for the prognosis of HCC (Fig. 3C–D). We also compared our signature with 10 other established prognostic signatures, including the Zhao signature [27], Liu signature [28], Fu signature [29], Wang signature[30], Zhigang signature [31], Yang signature [32], Liang signature [33], Tang signature [34], Li signature [35], and Tian signature [36]. Interestingly, our TRPS had the highest C-index compared with these 10 established prognostic signatures, suggesting that our TRPS had a relatively better performance in predicting the prognosis of HCC than some of the other signatures (Fig. 3E). Our TRPS had better performance when predicting overall survival of more than 5 years (Fig. 3F).

### Correlation analysis between TRPS and the immune microenvironment

**Fig. 4:**
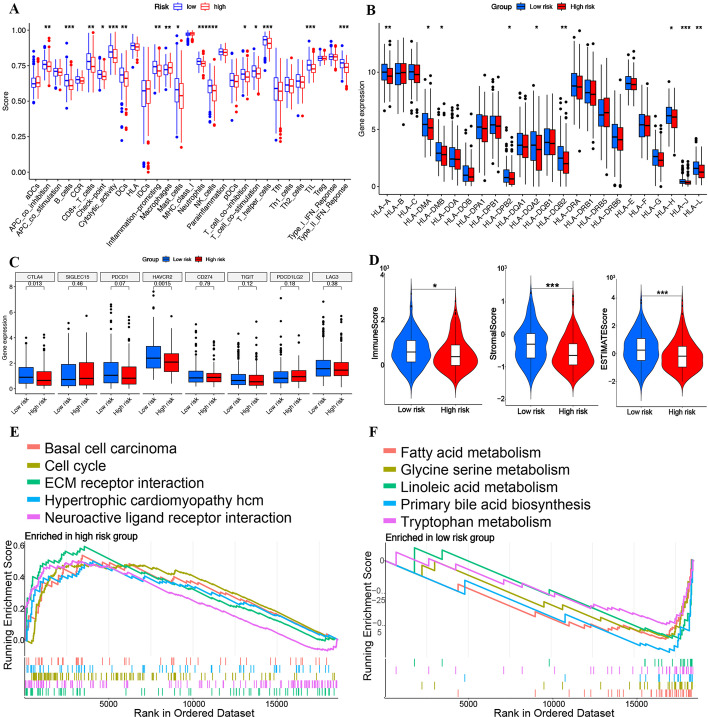
Immune microenvironment landscape and functional enrichment of the high- and low-risk groups. **A** The score of immune-related functions and components in the high- and low-risk groups. **B–C** The expression of HLA-related genes and immune checkpoints in the high- and low-risk groups. **D** The stromal score, immune score, and ESTIMAE score in the high- and low-risk groups. **E**–**F** Functional enrichment items in the high- and low-risk groups. *P < 0.05, **P < 0.01, ***P < 0.001

As T cells play a vital role in the immune microenvironment, we then explored the correlation between TRPS and the immune microenvironment. As shown in Fig. 4A, low risk was correlated with a higher score for most immune-related components, including B cells, CD8 + T cells, cytolytic activity, mast cells, NK cells, costimulation T cells, Th2 T cells and type II IFN response. Moreover, the expression of approximately half of the HLA-related genes was higher in the low-risk group than in the high-risk group (Fig. 4B). As shown in Fig. 4C, a significant difference in the expression of CTLA4 and HAVCR2 was obtained between the high-risk group and the low-risk group. Moreover, HCC patients in the low-risk group had a higher stromal score, immune score and ESTIMAE score than those in the high-risk score group (Fig. 4D). These results suggested that the low-risk subgroup may be an immune-hot subtype.

### The difference between the two groups in functional enrichment

GSEA revealed that the high-risk group was correlated with basal cell carcinoma, cell cycle, ECM-receptor interaction, neuroactive ligand‒receptor interaction and hypertrophic cardiomyopathy (HCM) (Fig. 4E). The low-risk group was correlated with glycine, serine and threonine metabolism, beta-alanine metabolism, fatty acid metabolism, and tryptophan metabolism (Fig. 4F). These results suggested that the low-risk group was significantly correlated with tumor metabolism.

### TRPS-related risk score-based treatment strategy for HCC

**Fig. 5:**
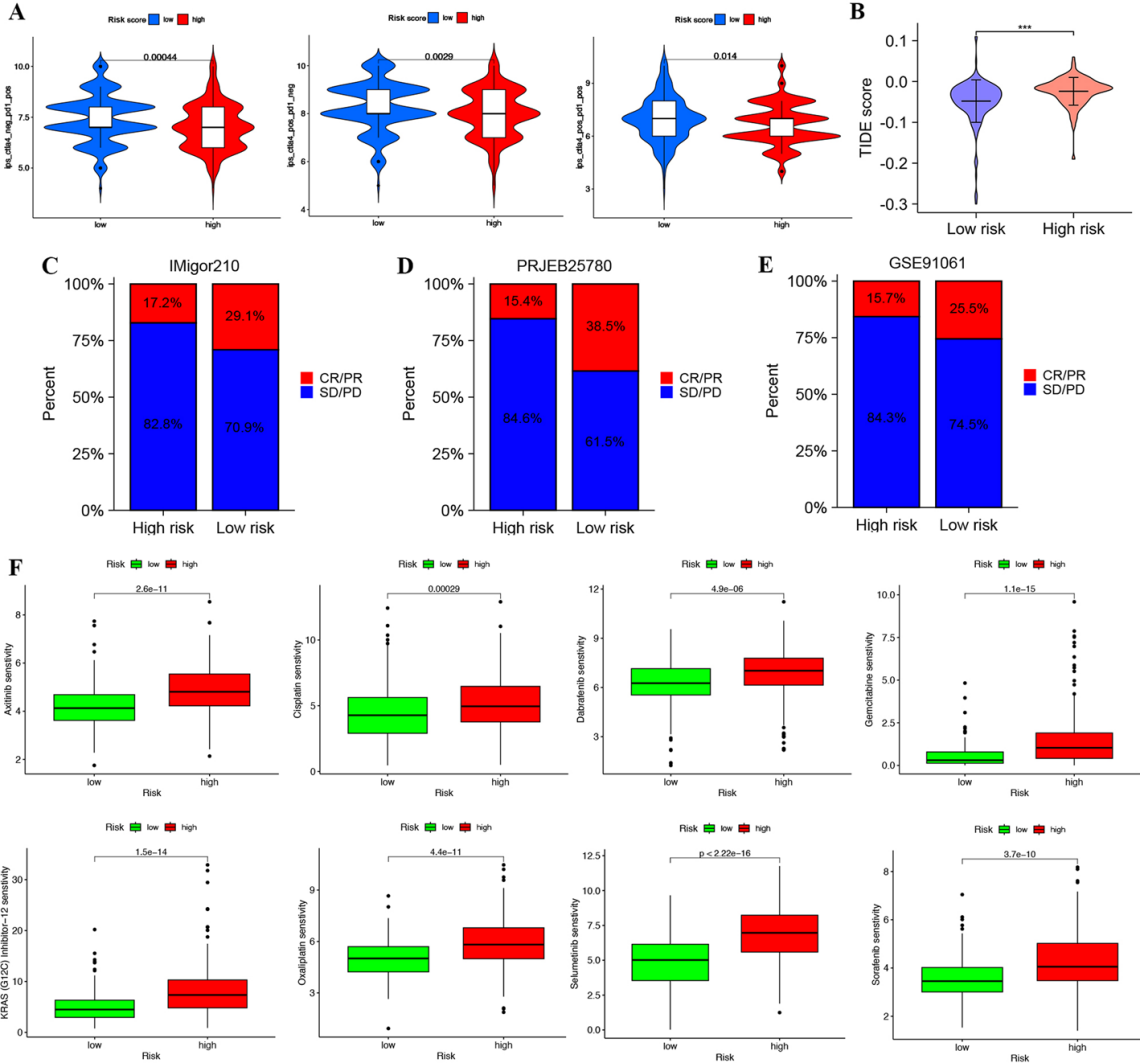
T-cell-related prognostic signature (TRPS)-based treatment strategy for HCC. **A** The immunophenoscore in the high- and low-risk groups. **B** The TIDE score in the high- and low-risk groups. The percentage of SD/PD and CR/PR response subtypes in patients with different risk scores in the IMigor210 cohort (**C**), PRJEB25780 cohort (**D**) and GSE91061 cohort (**E**). **F** The IC50 values of axitinib, cisplatin, dabrafenib, gemcitabine, KRAS inhibitor, oxaliplatin, selumetinib, and sorafenib in the high- and low-risk groups. *P < 0.05, **P < 0.01, ***P < 0.001

The above results suggested that the TRPS-related low-risk subgroup in HCC may be an immune-hot subtype. The TIDE score and IPS were good indicators for the prediction of immunotherapy response. A higher IPS and lower TIDE score indicated better sensitivity to immunotherapy. In our study, HCC patients in the low risk score group had a higher anti-PD 1 IPS, anti-CTLA4 IPS and anti-PD 1 and CTLA4 IPS than those in the high risk score group (Fig. 5A, all *p* < 0.05). Moreover, we also found that HCC patients in the low risk score group had a lower TIDE score than those in the high risk score group (Fig. 5B, p < 0.001). Thus, HCC in the low risk score group may be more sensitive to immunotherapy. Owing to the relatively few cases in the HCC immunotherapy database (GSE140901), we selected three datasets with follow-up information on the treatment effect to further verify the above results, including GSE91061 (n = 39, anti-CTLA4 and anti-PD1 therapy), PRJEB25780 (n = 78, anti-PD1 therapy) and IMigor210 (n = 298, anti-PD1 therapy). The response subtype was divided into two groups, PR/CR and SD/PD. The high TRPS-related risk score patients had a higher percentage of SD/PD, and CR/PR occurred more frequently in patients with low TRPS-related risk scores in the IMigor210 cohort (Fig. 5C), PRJEB25780 cohort (Fig. 5D) and GSE91061 cohort (Fig. 5E). These data revealed that HCC in the low risk score group may be more sensitive to immunotherapy than HCC in the high risk score group. We then compared the IC50 values of common drugs for chemotherapy and targeted therapy between the high- and low-risk score groups. As expected, HCC in the low risk score group had lower IC50 values for axitinib, cisplatin, dabrafenib, gemcitabine, KRAS inhibitor, oxaliplatin, selumetinib, and sorafenib (Fig. 5F). Thus, HCC in the low risk score group may be more sensitive to chemotherapy and targeted therapy than that in the high risk score group.

### Construction of a nomogram based on the TRPS

**Fig. 6:**
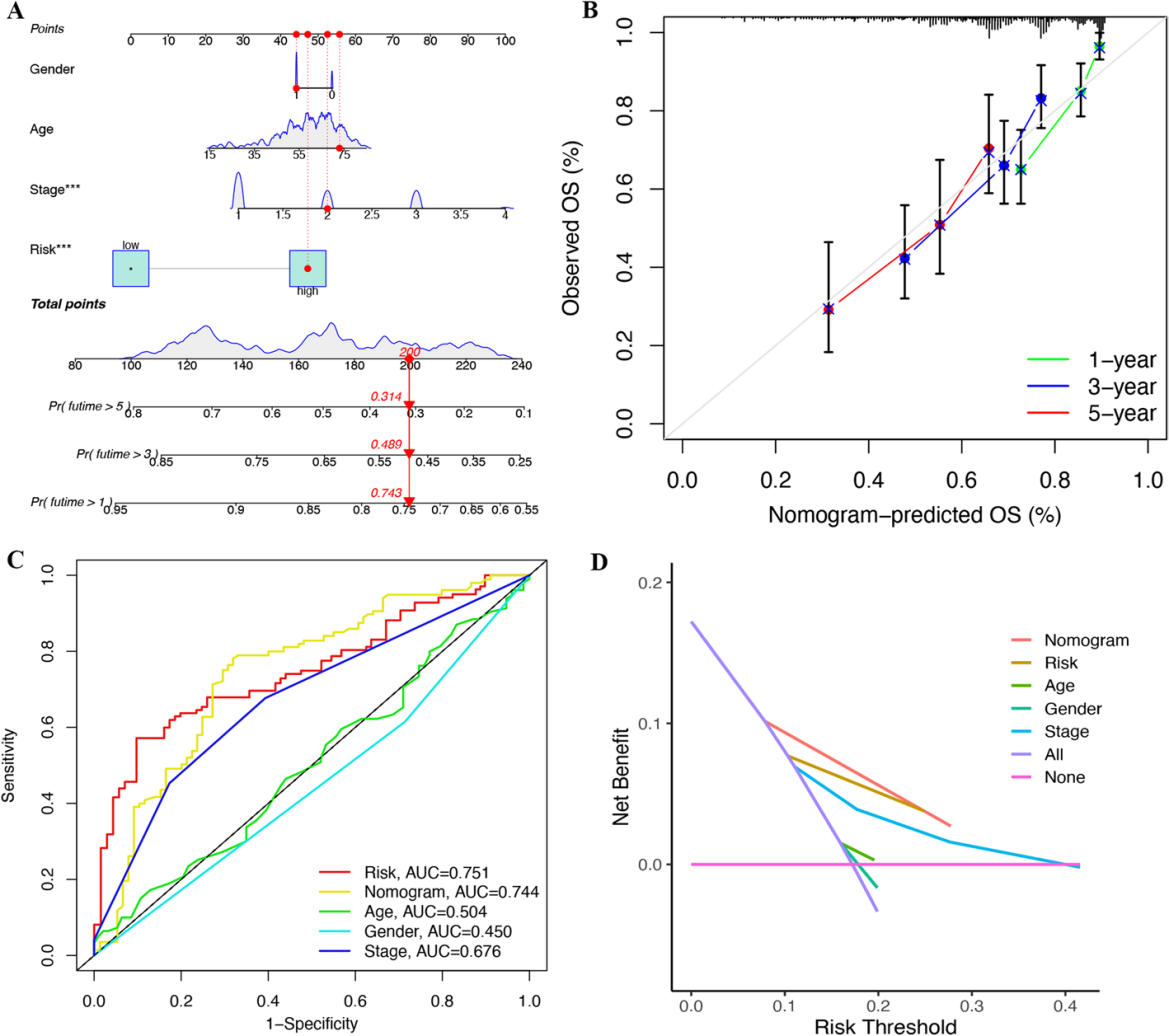
Construction of a nomogram based on the T-cell-related prognostic signature (TRPS) **A** A nomogram including clinical stage and TRPS predicting 1-year, 3-year and 5-year overall survival. **B** Calibration plots demonstrated that the actual 1-year, 3-year and 5-year survival times were highly consistent with the predicted survival times. **C** ROC curve comparing the predictive value of the nomogram, risk score and clinical parameters. **D** DCA curve revealed that this nomogram had a high potential for clinical application.

Based on the results of univariate and multivariate Cox regression analyses (Fig. 3C–D), we included clinical stage and TRPS in the construction of a nomogram (Fig. 6A). Calibration plots demonstrated that the actual 1-year, 3-year and 5-year survival times were highly consistent with the predicted survival times (Fig. 6B). Moreover, further ROC curve and DCA curve analyses revealed that this nomogram had high potential for clinical application (Fig. 6C–D).

## Discussion

With the development of cancer immunotherapy, an increasing number of markers to predict immunotherapy response have been identified. Increasing evidence has highlighted the vital role of the TME in the efficacy of cancer immunotherapy [37]. The development of scRNA-seq technologies has provided a potential way for researchers to explore the molecular characteristics of tumor-infiltrating immune cells in the TME. As one of the important mediating cells, T cells play a vital role in tumor immunotherapy [38]. Moreover, T-cell-based immunotherapy has elicited promising responses in malignancies, melanoma, and lung cancer [39]. However, reliable biomarkers based on T cells for immunotherapy response and prognosis of HCC are still rare.

In our study, scRNA-seq analysis identified 181 cell marker genes in two HCC samples. Further LASSO regression analysis screened 13 T-cell-related genes for the construction of a prognostic signature, including IL7R, BATF, PRDX1, HSPA8, AHSA1, RGS2, DYNLL1, CHORDC1, NUDC, OAZ1, PER1, ZC3HAV1, and CDV3. Many studies have highlighted the important role of these genes in the activity of T cells. The cytokine receptor IL-7R is critical for T-cell development, differentiation, generation and maintenance of memory T cells [40]. The cooperation of BATF and IRF4 could again exhaust tumor-infiltrating CAR T cells [41]. HSPA8 and ICAM-1 can act as damage-induced mediators of γδ T-cell activation [42]. Stress hormone signaling inhibits Th1 polarization in a CD4 T-cell-intrinsic manner via mTORC1 and PER1 [43].

Further study revealed that our TRPS could serve as a powerful predictive tool for the prognosis of HCC in TCGA and GSE14520 cohorts. Many prognostic signatures have been developed for HCC. Zhao et al. developed an amino acid metabolism-related signature for the prediction of HCC patients [27]. Based on six genes, another signature was developed to predict the OS rate of HCC [28]. The pyroptosis-related signature could serve as a prognostic biomarker for HCC and predict immune infiltration [29]. Based on the metabolic rate-limiting enzyme prognostic signature, clinicians may evaluate the prognosis and therapy response of HCC [30]. A novel five-gene signature could predict the OS rate of HCC [31]. Using the LASSO algorithm, Yang et al. developed a macrophage-related signature that could predict the clinical outcome of HCC [32]. To explore the role of ferroptosis in the prognosis of HCC, some researchers constructed a ferroptosis-related lncRNA signature for HCC [33]. A five-cholesterol metabolism-related gene signature could predict the prognosis of HCC patients [34]. Due to the vital role of m6A methyltransferase in HCC, Li et al. constructed a prognostic signature based on m6A methyltransferase-related lncRNAs that could predict the immunotherapy response of HCC [35]. Another signature constructed by CDC20, TOP2A, RRM2, UBE2C and AOX1 could predict the prognosis of HCC patients [36]. Compared with these 10 established prognostic signatures, TRPS had a higher C-index, suggesting a better performance of TRPS in predicting the prognosis of HCC. The data demonstrated that low risk was correlated with higher scores for some immune cells, HLA-related genes and immune checkpoints. Moreover, HCC patients in the low-risk group had a higher stromal score, immune score and ESTIMAE score than those in the high-risk score group. These results suggested that the low-risk subgroup may be an immune-hot subtype [44, 45]. The high score of immune cell infiltration can prevent tumor cell escape from immune surveillance and inhibit tumor progression, which may be one of the reasons why this subtype of HCC had a better overall survival. GSEA revealed that the high-risk groups were significantly associated with the biological processes of the cell cycle pathway. Hence, the inferior prognosis of HCC patients with high risk scores may be partly attributed to the abnormal regulation of the cell cycle, which is intimately linked to tumor proliferation and progression.

Moreover, the current study may provide a treatment strategy based on the TRPS-related risk score for HCC. We found that patients with a high TRPS risk score had a higher TIDE score and lower immunophenoscore. The high-risk score patients had a higher percentage of SD/PD, and CR/PR occurred more frequently in patients with low TRPS-related risk scores in the IMigor210, PRJEB25780 and GSE91061 cohorts. The TIDE score [46] and immunophenoscore [26] were good predictors of the immune response. A high score and low immunophenoscore indicated low sensitivity to immunotherapy. Thus, the current result may suggest that HCC patients in the low-risk group were more likely to benefit from immunotherapy. TRPS might act as a reliable biomarker for predicting immunotherapy response.

Some limitations should be mentioned in our study. Only two single-cell datasets of HCC were used in our study, and they could not represent all other HCC patients. The expression and prognosis of TRPS should be detected using clinical tissues. Moreover, the performance of the TRPS in risk evaluation in HCC was better than that of age and sex but not clinical stage, which was different from the results of the TCGA dataset. It would be better to verify these results using more datasets. It would be better to compare our TRPS with more developed signatures for HCC.

## Conclusion

Our study constructed and validated a novel T-cell-related prognostic signature by integrated analysis of single-cell and bulk RNA sequencing, which could serve as a reliable biomarker for predicting prognosis and immunotherapy. Our study may provide novel insight into the role of immune cell marker genes in the prognosis and immunotherapy response of HCC patients.

## Supplementary Information

**Additional file 1: Table S1.** The details and characteristics of singe cell HCC cases.

**Additional file 2: Table S2.** The Details and characteristics of TCGA and GSE14520 cohort.

**Additional file 3: Table S3.** Cell markers identified by single-cell sequencing analysis.

**Additional file 4: Figure S1.** The CNV landscape of T cell markers in HCC.

**Additional file 5: Figure S2.** SNV landscape and prognostic value of T cell markers in HCC. (A-B) The SNV landscape of T cell markers in HCC. (C) Potential prognostic biomarkers identifying by univariate cox regression analysis.

**Additional file 6: Figure S3.** Construction and validation of a T cell-related prognostic signature (TRPS) for HCC. (A-B) The coefficient and partial likelihood deviance of TRPS. (C-D) Risk scores distribution, patients’ survival status, gene expression heatmap of TRPS in TCGA cohort and GSE14520cohort.

## Data Availability

The analyzed datasets generated during the study are available from the corresponding author on reasonable request.
