# Supplementary material for: A T-cell-related signature for prognostic stratification and immunotherapy response in hepatocellular carcinoma based on transcriptomics and single-cell sequencing
Source: BMC Bioinformatics. 2023 May 25;24:216. doi: 10.1186/s12859-023-05344-7 (PMC10210368; doi:10.1186/s12859-023-05344-7)
Supplement: Supplementary file 1 — Additional file 1: Table S1. The details and characteristics of singe cell HCC cases. [file 12859_2023_5344_MOESM1_ESM.docx]

**Supplementary Table 1. The Details and characteristics of singe cell HCC cases.**

| Patient ID | Gender | Age | Tumor size(cm) | HBV | Tumor grade | TNM stage | Lymphnode  metastasis | Distant metastasis | Sample type | American Joint Committee on Cancer Publication version type |
| --- | --- | --- | --- | --- | --- | --- | --- | --- | --- | --- |
| GSM4955419 | M | 50 | 3 | Y | 2-3 | II | N | N | Primary | 7th |
| GSM4955421 | M | 76 | 3.5 | Y | 3 | II | N | N | Primary | 7th |
