# Supplementary material for: A T-cell-related signature for prognostic stratification and immunotherapy response in hepatocellular carcinoma based on transcriptomics and single-cell sequencing
Source: BMC Bioinformatics. 2023 May 25;24:216. doi: 10.1186/s12859-023-05344-7 (PMC10210368; doi:10.1186/s12859-023-05344-7)
Supplement: Supplementary file 2 — Additional file 2: Table S2. The Details and characteristics of TCGA and GSE14520 cohort. [file 12859_2023_5344_MOESM2_ESM.docx]

**Supplementary Table 2. The Details and characteristics of TCGA and GSE14520 cohort.**

| **Covariates** | **TCGA** | **GSE14520** |
| --- | --- | --- |
| **Age** | 58.85(21-77) | 55(21-57) |
| **Gender** |  |  |
| Male | 249 | 189 |
| Female | 121 | 32 |
| **Stage** |  |  |
| Stage I | 175 | 94 |
| Stage II | 86 | 78 |
| Stage III | 86 | 49 |
| Stage IV | 5 | 0 |
| Unknow | 18 | 0 |
| **T** |  |  |
| T1 | 187 |  |
| T2 | 84 |  |
| T3 | 85 |  |
| T4 | 13 |  |
| Unknow | 1 |  |
| **N** |  |  |
| N0 | 257 |  |
| N1 | 4 |  |
| Unknow | 109 |  |
| **M** |  |  |
| M0 | 272 |  |
| M1 | 4 |  |
| Unknow | 94 |  |
