# Supplementary figures and images for: A T-cell-related signature for prognostic stratification and immunotherapy response in hepatocellular carcinoma based on transcriptomics and single-cell sequencing
Source: BMC Bioinformatics. 2023 May 25;24:216. doi: 10.1186/s12859-023-05344-7 (PMC10210368; doi:10.1186/s12859-023-05344-7)

**Supplementary Figure 1. The CNV landscape of T cell markers in HCC.**


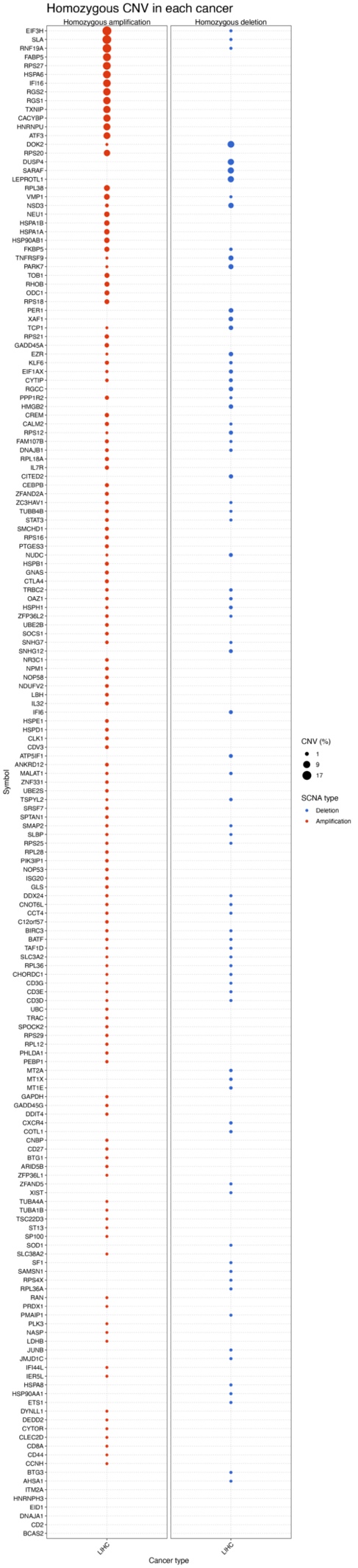

Supplement: Supplementary file 4 — Additional file 4: Figure S1. The CNV landscape of T cell markers in HCC. [file 12859_2023_5344_MOESM4_ESM.docx]
