# Supplementary material for: A T-cell-related signature for prognostic stratification and immunotherapy response in hepatocellular carcinoma based on transcriptomics and single-cell sequencing
Source: BMC Bioinformatics. 2023 May 25;24:216. doi: 10.1186/s12859-023-05344-7 (PMC10210368; doi:10.1186/s12859-023-05344-7)

**Supplementary Figure 2. SNV landscape and prognostic value of T cell markers in HCC.** (A-B) The SNV landscape of T cell markers in HCC. (C) Potential prognostic biomarkers identifying by univariate cox regression analysis.


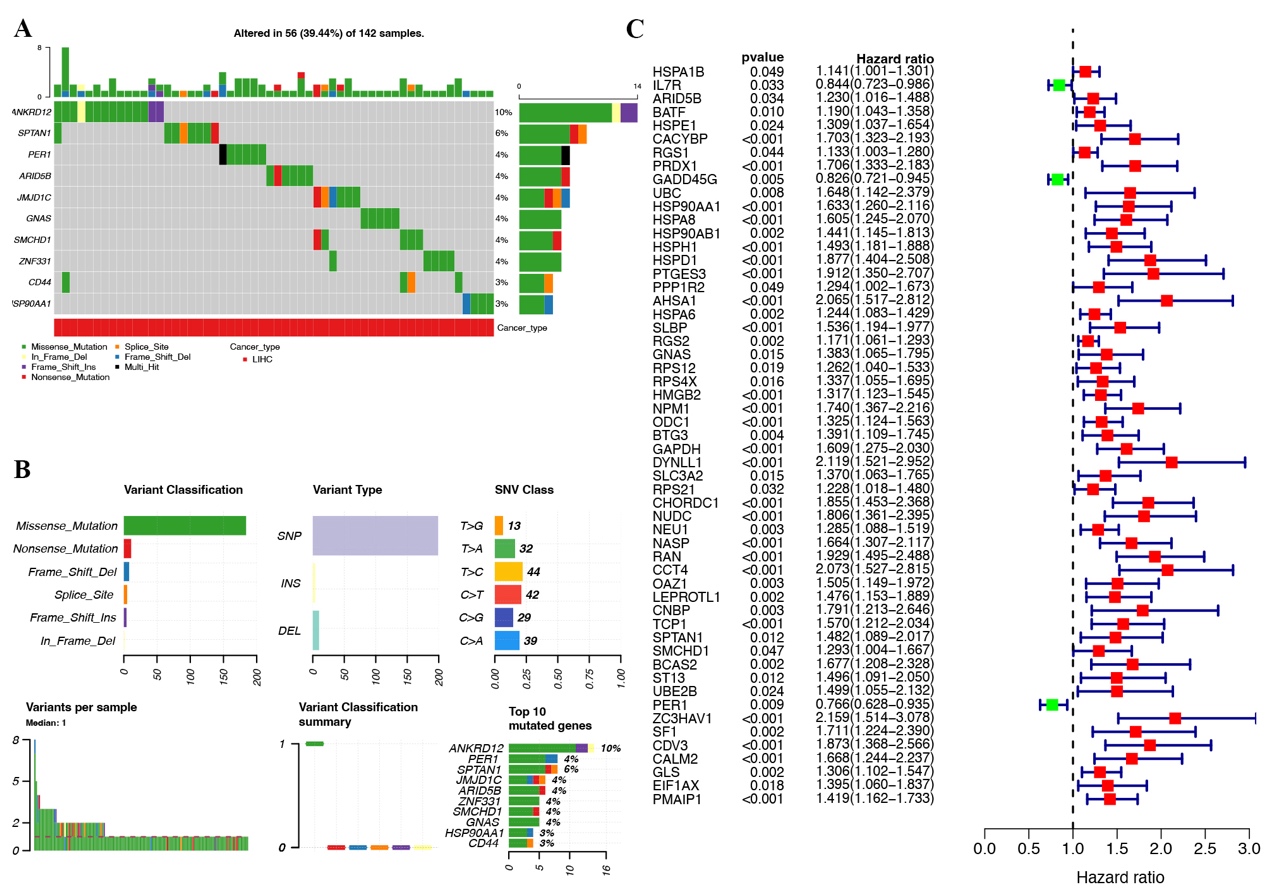

Supplement: Supplementary file 5 — Additional file 5: Figure S2. SNV landscape and prognostic value of T cell markers in HCC. (A-B) The SNV landscape of T cell markers in HCC. (C) Potential prognostic biomarkers identifying by univariate cox regression analysis. [file 12859_2023_5344_MOESM5_ESM.docx]
