# Supplementary material for: A T-cell-related signature for prognostic stratification and immunotherapy response in hepatocellular carcinoma based on transcriptomics and single-cell sequencing
Source: BMC Bioinformatics. 2023 May 25;24:216. doi: 10.1186/s12859-023-05344-7 (PMC10210368; doi:10.1186/s12859-023-05344-7)

**Supplementary Figure 3. Construction and validation of a T cell-related prognostic signature (TRPS) for HCC.** (A-B) The coefficient and partial likelihood deviance of TRPS. (C-D) Risk scores distribution, patients’ survival status, gene expression heatmap of TRPS in TCGA cohort and GSE14520cohort.


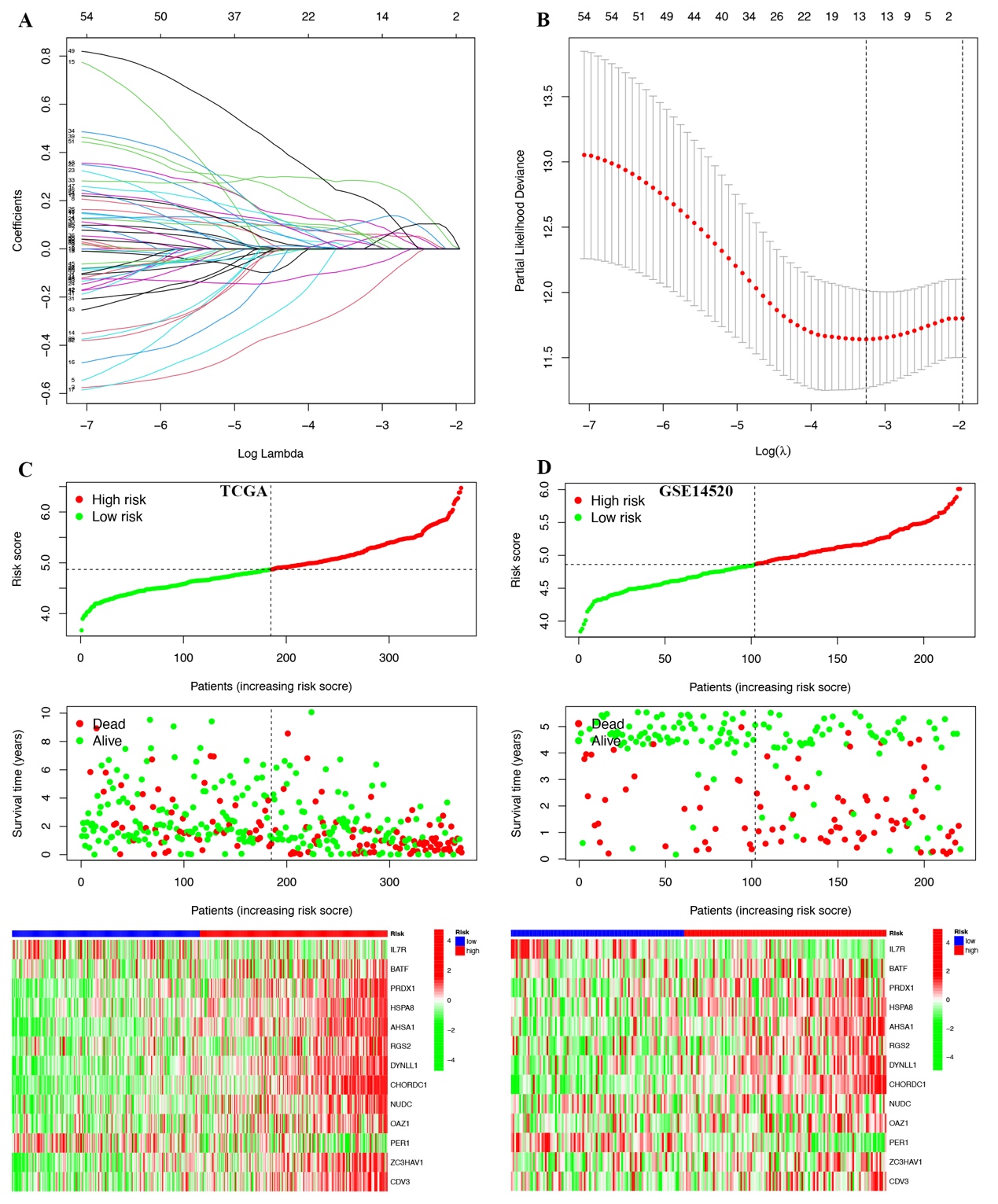

Supplement: Supplementary file 6 — Additional file 6: Figure S3. Construction and validation of a T cell-related prognostic signature (TRPS) for HCC. (A-B) The coefficient and partial likelihood deviance of TRPS. (C-D) Risk scores distribution, patients’ survival status, gene expression heatmap of TRPS in TCGA cohort and GSE14520cohort. [file 12859_2023_5344_MOESM6_ESM.docx]
